# Second Genome: Rhizosphere Microbiome as a Key External Driver of Nitrogen Use Efficiency in Maize

**DOI:** 10.3390/plants14233680

**Published:** 2025-12-03

**Authors:** Ping Luo, Lin Yang, Yonghui Zhu, Mao Liu, Yuanyuan He, Chengwei Liu, Wenzhu He

**Affiliations:** Crop Research Institute (Sichuan Germplasm Resources Center), Sichuan Academy of Agricultural Sciences, Chengdu 610066, China; luoping987@126.com (P.L.); ownmylife@163.com (L.Y.); yhzhu86@hotmail.com (Y.Z.); 19162803124@163.com (M.L.); yuanyuanhe@163.com (Y.H.); chengweiliu@163.com (C.L.)

**Keywords:** maize, nitrogen use efficiency, rhizosphere microbiome, multi-omics, molecular breeding

## Abstract

Improving nitrogen use efficiency (NUE) in maize is critical for reducing fertilizer inputs and mitigating environmental impacts. The rhizosphere microbiome—the plant’s “second genome”—plays a key role in nitrogen acquisition, assimilation, and signaling. This review synthesizes recent advances from multi-omics studies, functional validation, and field experiments, highlighting how maize roots recruit and coordinate microbial taxa, including diazotrophs, nitrifiers, organic nitrogen mineralizers, and growth-promoting bacteria, to enhance NUE under variable nitrogen availability. We integrate mechanistic insights into transporter-mediated nitrogen uptake, microbial regulation of root development and exudation, and host genetic determinants, such as *ZmC2*, *ZmSBT3*, and ZmNLP8, that influence microbiome assembly. Evidence from synthetic communities, isotope tracing, and host–microbiome association studies demonstrates that microbial contributions to plant nitrogen can be substantial and genetically modulated. Finally, we discuss microbiome-based interventions, including functional strain discovery, microbial fertilizers, biostimulants, and microbiome-assisted breeding, assessing their potential and limitations. We conclude by highlighting key challenges and proposing an integrative framework to guide microbiome-informed strategies for sustainable improvement of maize NUE.

## 1. Introduction

Nitrogen (N) fertilizers play a pivotal role in global crop production, yet their utilization efficiency is typically low, posing both economic and environmental challenges. In China, millions of smallholder farmers often apply excessive nitrogen fertilizers due to limited access to precise management practices, aiming to avoid yield losses. Between 1961 and 2016, nitrogen fertilizer application in China increased from ~4.3 million tons to 18.6 million tons [1,2]. However, approximately 50~70% of applied nitrogen is not assimilated by crops and is instead lost to the environment, contributing to soil acidification, water eutrophication, and greenhouse gas emissions [3,4]. Understanding how crops respond to dynamic soil nitrogen availability is therefore crucial for improving nitrogen use efficiency (NUE) and achieving sustainable agricultural production.

Maize (*Zea mays* L.) is the most widely cultivated cereal crop globally, accounting for approximately 35% of total cereal production and playing a critical role in human food and nutritional security. In 2020, the global maize cultivation area was ~197 million hectares, with nitrogen fertilizer consumption of ~109 million tons, a demand projected to rise to ~443 million tons by 2050 [5]. In China, maize serves not only as a staple, commercial, and feed crop but also as a major source of livestock feed for meat, eggs, and milk. Maize production has exceeded that of rice since 2012, making it the dominant cereal crop in China. According to the National Bureau of Statistics, total maize production reached 295 million tons in 2024, an increase of approximately 2.1% compared with 2023. Amid rising global nitrogen fertilizer demand and increasing environmental pressures, developing maize cultivars with high nitrogen use efficiency (NUE) is a key strategy to achieve both high yield and sustainable production. High-NUE varieties can maintain or even enhance yield under reduced nitrogen inputs, thereby improving resource use efficiency and mitigating ecological impacts. NUE is a complex trait regulated by multiple factors and is typically divided into nitrogen uptake efficiency (NUPE) and nitrogen utilization efficiency (NUTE) [6]. Recent studies in maize and model plants, including Arabidopsis thaliana and rice, have identified numerous NUE-associated regulators, such as nitrate and ammonium transporters, key nitrogen assimilation enzymes, and root developmental regulators [7,8,9]. In addition, root perception of and adaptive responses to spatial heterogeneity of soil nitrogen are considered central to the establishment of efficient nitrogen utilization.

Recent advances in high-throughput microbiome technologies have substantially deepened our understanding of the pivotal role played by rhizosphere microbial communities in regulating maize nitrogen uptake and utilization [10,11,12]. The intricate host–microbe nitrogen interaction network governs not only nitrogen absorption, transport, and assimilation but also its redistribution and signaling cascades within plant tissues [12]. These insights provide a conceptual framework for integrating molecular breeding with precision agronomic management to enhance maize nitrogen use efficiency in a sustainable manner. Historically, efforts to improve maize NUE have primarily focused on host-level genetic improvement, including the manipulation of nitrate and ammonium transporters, transcriptional regulators, and root system architecture [13,14,15]. However, emerging evidence suggests that the rhizosphere microbiome, often regarded as the “second genome” of plants, acts as a critical determinant of nitrogen acquisition, assimilation, and remobilization [10,16,17]. Through processes such as inorganic nitrogen transformation, organic nitrogen mineralization, modulation of nitrogen responsive signaling pathways, and alteration of root exudate profiles, rhizosphere microorganisms exert profound direct and indirect effects on plant nitrogen metabolism [18,19].

Elucidating the molecular mechanisms underpinning host–microbiome interactions will provide new avenues for breeding maize varieties with superior NUE [10]. Despite the remarkable progress achieved in recent years, realizing efficient and environmentally sustainable nitrogen management remains a formidable challenge. Future research should aim to establish a multi-tiered integrative framework that links mechanistic insights, microbiome functional exploration, breeding innovation, and field management practices [3,20,21]. Such a system-level approach aligning host nitrogen sensing, uptake, and utilization processes with optimized rhizosphere microbial functions will enable a precise synchronization between nitrogen supply and plant demand, thereby contributing to both global food security and sustainable agricultural development [16,22].

## 2. Transporter-Mediated Mechanisms of Efficient Nitrogen Uptake and Translocation in Maize

In recent years, extensive progress has been made in elucidating the mechanisms underlying plant nitrogen uptake and utilization, with research focusing primarily on nitrate and ammonium transporters, signaling networks, transcriptional regulation, and root system architecture. Plants mainly acquire nitrogen in the form of inorganic nitrate (NO_3_^−^) and ammonium (NH_4_^+^), although certain organic nitrogen sources—such as urea, peptides, and amino acids—can also be absorbed and metabolized. Urea, owing to its high nitrogen content, is widely used in agricultural production. In soils, it is hydrolyzed by urease to release ammonium, which is subsequently converted to nitrate through microbial nitrification. In arid and semi-arid ecosystems, where nitrification rates are relatively high and nitrate leaching is limited, soil nitrate levels generally exceed those of ammonium. Maize, a nitrogen-demanding crop, primarily absorbs nitrogen in the form of nitrate through its root system. Once taken up, nitrate can either be reduced and assimilated in the roots or translocated via the xylem to aerial parts, where it contributes to nitrogen metabolism or serves as a temporary storage pool. Numerous studies have demonstrated that modulating the expression and activity of nitrate and ammonium transporters significantly enhances nitrogen uptake and partitioning efficiency, thereby improving overall nitrogen use efficiency [23,24]. Over the course of evolution, plants have developed two distinct nitrate transport systems to adapt to fluctuating soil nitrogen availability: the high-affinity transport system (HATS) and the low-affinity transport system (LATS). HATS operates effectively under low external nitrate concentrations, whereas LATS predominates under high nitrate conditions, ensuring a dynamic and efficient response to environmental nitrogen fluctuations [25].

### 2.1. Molecular Regulation of Nitrate (NO_3_^−^) Transporters and Nitrogen Assimilation

In plants, members of the nitrate transporter 1/peptide transporter (NRT1/PTR, also known as NPF) family primarily function in the LATS, whereas NRT2 family proteins are responsible for HATS, collectively enabling plants to adapt to the dynamic fluctuations of nitrogen availability in soil [26,27]. In maize, four NRT1.1 homologs have been identified, which can be phylogenetically classified into three evolutionary clades [23,28]. Functional characterization in heterologous expression systems revealed that ZmNPF6.6/ZmNRT1.1B exhibits a dual-affinity transport capacity, facilitating nitrate uptake under both low and high external nitrate concentrations. In contrast, ZmNPF6.4/ZmNRT1.1A displays low-affinity transport activity only [29]. This difference is attributed to structural variation between the two proteins, particularly the presence of the His362 residue in ZmNRT1.1B, which is essential for nitrate binding and transport but absent in ZmNRT1.1A [29]. Further functional validation demonstrated that ZmNRT1.1B mediates both root uptake and root–shoot translocation of nitrate. Overexpression of this gene in modern maize hybrids significantly increased yield even under reduced nitrogen fertilizer input.

The putative high-affinity transporters ZmNRT2.1 and ZmNRT2.2 are transcriptionally responsive to the availability of both nitrate and carbon sources—particularly sugars [30]. Their uptake activity likely depends on interaction with the partner protein ZmNAR2.1 [31]. Overexpression of ZmNRT2.1 in tobacco enhanced root growth under calcium-deficient conditions, regardless of nitrate concentration [32]. Although the ZmNRT2 family has been implicated in nitrate uptake, its regulatory mechanisms, spatiotemporal expression patterns, and precise contribution to maize NUE remain to be systematically elucidated. Beyond classical nitrate transporters and reductases, several other genes have been identified to modulate nitrate accumulation and downstream nitrogen metabolism. In maize natural populations, a key gene, *ZmNCRG1*, encoding a chloroplast-localized ferredoxin ZmFd4, was found to be significantly associated with shoot nitrate content. ZmFd4 interacts directly with nitrite reductase (ZmNiR) and forms dimers with its homolog ZmFd9, thereby regulating ZmNiR enzymatic activity and influencing nitrate reduction and subsequent nitrogen assimilation processes [33]. Moreover, the transcription factor NIN-like protein 8 (ZmNLP8) plays a pivotal role in nitrogen signaling. Loss-of-function mutants exhibit premature senescence and more than 70% reduction in grain yield under sufficient nitrogen conditions, whereas overexpression lines display superior growth performance under fluctuating nitrate supply [34]. Mechanistically, ZmNLP8 binds to nitrate-responsive elements (NREs) to activate the transcription of ZmNiR1.2, promoting nitrate assimilation and enhancing nitrogen use efficiency.

**Figure 1:**
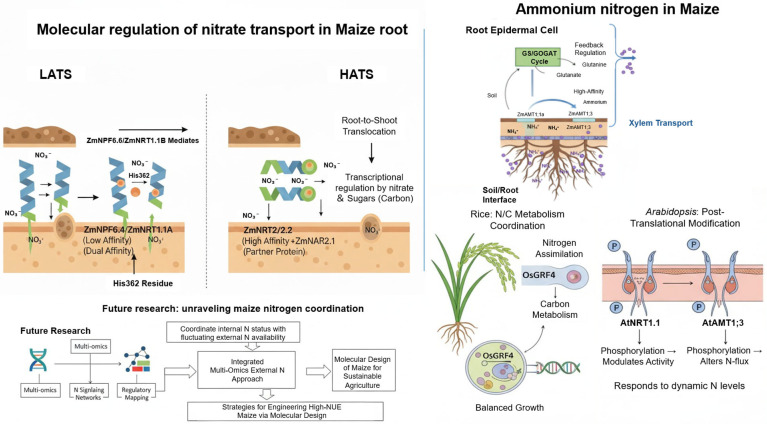
Regulatory network of nitrogen uptake and assimilation in maize. Nitrate (NO_3_^−^) and ammonium (NH_4_^+^) uptake is mediated by low- and high-affinity transporters (ZmNPF/NRT1, ZmNRT2, ZmAMT1), while downstream assimilation involves ferredoxin-mediated nitrite reduction (ZmFd4/ZmFd9-ZmNiR) and transcriptional regulation by ZmNLP8 and *MADS26*. These interconnected modules integrate nitrogen sensing, signaling, and metabolism, shaping genotype-specific nitrogen use efficiency.

Recent time-series transcriptomic analyses further revealed genotype-dependent variation in nitrate responsiveness among maize inbred lines. The MADS-box transcription factor *MADS26* was identified as a key regulator in nitrate uptake and assimilation. Overexpression of *MADS26* conferred enhanced sensitivity to chlorate and markedly increased nitrate uptake and metabolic activity, suggesting that this factor modulates nitrogen assimilation through regulation of nitrogen sensing and signaling pathways [35]. Collectively, these transcriptional and signaling modules constitute a multilayered regulatory network governing nitrogen metabolism in maize, offering novel insights into the molecular basis of host nitrogen use efficiency (Figure 1).

### 2.2. Ammonium (NH_4_^+^) Uptake and Regulatory Mechanisms in Maize

Compared with other gramineous crops, maize possesses a particularly strong capacity for nitrogen uptake and superior energy-use efficiency, displaying a marked preference for ammonium as the nitrogen source [36]. Two high-affinity ammonium transporters, ZmAMT1;1a and ZmAMT1;3, have been identified as key components mediating ammonium acquisition in maize roots. These genes are predominantly expressed in the epidermal cells of the root apex and are responsible for high-efficiency ammonium uptake under nitrogen-limited conditions [36]. Interestingly, their transcriptional regulation does not respond directly to external ammonium levels but is modulated through glutamine-mediated feedback, indicating a fine-tuned endogenous control mechanism within the nitrogen assimilation network [37]. Overexpression of ZmAMT1;1a has been reported to enhance ammonium uptake and improve plant growth under low-nitrogen conditions [37], suggesting its potential utility in molecular breeding for high nitrogen-use efficiency.

Insights from other plant systems further highlight the complexity of nitrogen uptake regulation. In rice, *OsGRF4* (Growth-Regulating Factor 4) acts as a pivotal transcriptional regulator that coordinates nitrogen assimilation with carbon metabolism, promoting NUE and balanced growth [38]. Similarly, in Arabidopsis thaliana, post-translational modifications, such as phosphorylation of AtNRT1.1 and AtAMT1;3, modulate transporter activity and nitrogen flux in response to dynamic nutrient availability [39,40,41,42]. Despite these advances, the regulatory framework underlying ammonium transport and signaling in maize remains insufficiently characterized. Future research integrating multi-omics approaches, nitrogen signaling networks, and regulatory element mapping will be essential to uncover how maize coordinates internal nitrogen status with external nitrogen availability. Such efforts will deepen our understanding of nitrogen acquisition strategies in cereal crops and facilitate the molecular design of maize varieties with enhanced NUE for sustainable agriculture (Figure 1).

## 3. The Rhizosphere Microbiome in Plant Growth and Developmental Regulation

The term “microbiome” refers to the complex ecological system formed by microbial communities within a specific habitat and their surrounding environment, encompassing microbial community composition, metabolic activity, and multi-layered interactions with the environment. In contrast, “microbiota” denotes the actual assemblage of microbial organisms present within the community. Virtually all eukaryotic organisms coexist with diverse microbiota, which exert profound influences on host physiology. Although a small fraction of these microbes acts as pathogens or obligate symbionts, producing strong positive or negative effects on host fitness, the majority function as commensal or mutualistic microbes. These microbes subtly but persistently modulate host gene expression, metabolic states, and phenotypic traits. For instance, transcriptomic analyses across different intestinal regions of germ-free versus conventionally raised mice revealed that thousands of host genes are regulated by the composition and activity of the microbiome [43]. In plant systems, studies using synthetic communities (SynComs) in Arabidopsis thaliana have demonstrated analogous transcriptional responses, further confirming the ubiquity and significance of microbiome-mediated regulation of host gene networks [44,45]. Collectively, these findings underscore the central role of the microbiome as a key modulator of host phenotypes and their interactions with the environment.

The study of Plant Growth-Promoting Rhizobacteria (PGPRs) predates the formal conceptualization of the plant microbiome [46,47,48,49,50]. In natural environments, exogenous PGPRs must compete with resident rhizosphere microbes, such that single-strain effects observed under controlled laboratory conditions are often not fully recapitulated in the field. PGPRs can modulate plant growth and development by producing or modulating phytohormones, including Auxins, Cytokinins, Abscisic acid, Ethylene, and Gibberellins [51]. However, phytohormone levels require precise regulation, as excessive or ectopic accumulation can inhibit growth. For example, certain members of the microbial community may suppress root elongation through auxin production. Notably, *Variovorax* strains in the rhizosphere can degrade auxin metabolites, counteracting the negative effects of other community members on root phenotypes, thereby illustrating that even a single microbial genus can substantially influence host traits [52]. Beyond developmental regulation, the microbiome enhances plant performance under stress conditions, such as nutrient limitation. This is primarily achieved by converting soil mineral nutrients into bioavailable forms and facilitating their allocation between root and shoot tissues, thereby supporting overall plant growth and productivity [53].

## 4. Rhizosphere Microbiome-Mediated Promotion of Nitrogen Uptake in Maize

Extensive studies have demonstrated that inoculation with nitrogen-fixing microorganisms can significantly promote plant growth, primarily by providing the host with bioavailable nitrogen [54]. Diverse plant species, including maize, establish intimate interactions with soil microbial communities, which play pivotal roles in nutrient acquisition and growth regulation [55,56]. Plant genotypes can modulate the composition of rhizosphere microbiota through root exudates, which act as both signals and carbon sources, selectively attracting soil microbes to colonize the root surface or rhizosphere [57]. Concurrently, the plant immune system determines which microorganisms successfully establish and persist within the root environment. In turn, beneficial rhizosphere microbes can activate host signaling pathways and metabolic networks, enhancing nutrient uptake, stress resilience, and overall growth and development [58,59].

**Figure 2:**
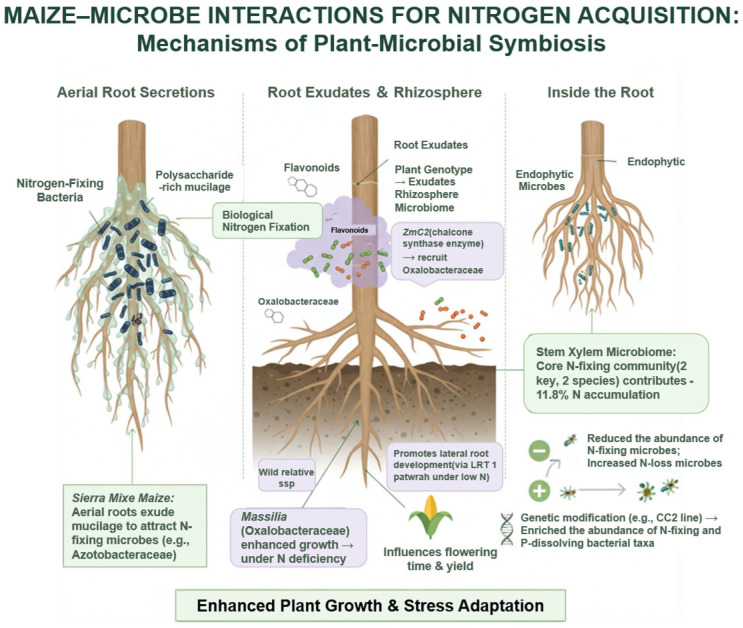
Rhizosphere microbiome-mediated promotion of nitrogen uptake in maize. Maize roots secrete carbohydrates and flavonoids that recruit and shape rhizosphere microbial communities, including nitrogen-fixing bacteria, which enhance nitrogen acquisition, root development, and plant growth under nitrogen-limited conditions.

Although maize lacks intrinsic nitrogen-fixing ability, accumulating evidence indicates that it can adjust rhizosphere microbial composition to cope with nitrogen-limited conditions [60]. Maize can associate with various diazotrophic bacteria, thereby indirectly utilizing atmospheric nitrogen to support plant growth [61,62]. Field experiments in nitrogen-poor soils in Mexico demonstrated that the aerial roots of the local maize landrace Sierra Mixe secrete polysaccharide-rich mucilage, which recruits and stabilizes nitrogen-fixing bacteria, facilitating biological nitrogen fixation [62]. Interestingly, the wild relative Zea mays ssp. *Mexicana* retains a similar capability, albeit at a reduced secretion level, suggesting that this nitrogen-fixing interaction represents a conserved strategy originating from early domestication. Moreover, carbohydrate-rich aerial root mucilage has been detected in certain modern inbred lines (Figure 2) [63], implying that this ancient trait persists partially in contemporary germplasm. Genome-wide association studies have identified *ZmSBT3*, a key regulatory factor within the Bacillus subtilis family, as a negative regulator of aerial root mucilage secretion; knockout of this gene significantly increases plant biomass and total nitrogen accumulation, highlighting the potential of harnessing microbe-mediated interactions to enhance nitrogen use efficiency under nitrogen-limited conditions [63].

In addition to carbohydrates, maize roots secrete diverse flavonoid secondary metabolites that play critical roles in recruiting and modulating rhizosphere microbial communities. Flavonoids synthesized by chalcone synthase *ZmC2* facilitate the colonization of Oxalobacteraceae members in the rhizosphere [64]. The enrichment of these bacteria, in turn, promotes lateral root development through LRT1-mediated developmental signaling pathways, enhancing nitrogen uptake under nitrogen-limited conditions and acting as a central regulator of root branching and environmental responsiveness [64,65]. Furthermore, the abundance of the genus *Massilia* negatively correlates with soil nitrogen content; under low nitrogen conditions, inoculation with Massilia promotes the growth of *zm00001d048945* mutants in both roots and shoots [60], indicating that Massilia represents a key ecological driver of rhizosphere microbial assembly under nitrogen deficiency. Additional studies suggest that Massilia can indirectly enhance maize yield by modulating the expression of genes associated with flowering time (Figure 2) [66].

Remarkably, recent findings indicate that the maize stem xylem harbors microbiota analogous to the human gut microbiome, selectively colonizing core microbiota. A synthetic community comprising two core nitrogen-fixing bacteria (active diazotrophs) and two auxiliary strains (supporting nitrogen fixation) can facilitate biological nitrogen fixation, contributing approximately 11.8% of stem nitrogen accumulation in maize [10]. However, modern breeding for improved nitrogen use efficiency may alter rhizosphere microbiome composition. Evidence suggests that some improved germplasm exhibits reduced abundance of potential nitrogen-fixing or nitrogen acquisition-promoting taxa, while communities associated with nitrogen loss are relatively enriched [67,68]. Genetic engineering offers a strategy to reconstruct rhizosphere microbiota favorable for plant nitrogen utilization; for instance, glyphosate-tolerant transgenic line CC2 shows increased abundance of nitrogen-fixing and phosphate-solubilizing bacterial groups (Figure 2) [69].

## 5. Multi-Omics Dissection of Rhizosphere Microbiome–Nitrogen Interactions

Recent advances in multi-omics approaches are transforming research on NUE from single-factor analyses to integrative, system-level investigations. In maize, the combined application of metagenomics, transcriptomics, metabolomics, and proteomics has revealed critical roles of rhizosphere microbes in nitrogen acquisition, assimilation, and cycling. For instance, ^15^N isotope tracing coupled with metagenomics identified 25 core bacterial taxa in maize stem xylem, and a synthetic community of active nitrogen-fixing and auxiliary bacteria was shown to contribute ~11.8% of stem nitrogen, highlighting the potential for symbiotic nitrogen fixation in maize [10]. Population-level studies further indicate that host genotype strongly shapes rhizosphere microbial composition under nitrogen-limited conditions, emphasizing the central role of host genetics in microbiome assembly and adaptive responses [11]. Integrating amplicon sequence variants (ASVs) and host single-nucleotide polymorphisms (SNPs) into genome-wide selection models has substantially improved prediction accuracy for nitrogen-related traits such as yield and NUE, demonstrating the potential of host–microbiome-assisted breeding [70].

Metabolomic profiling reveals that root exudates, including flavonoids and organic acids, act as key signals for recruiting nitrogen-responsive microbes, thereby enhancing nutrient acquisition. These exudate profiles are dynamically regulated by nitrogen availability, crop rotation, and intercropping, and closely correlate with microbial community structure and nitrogen-cycling functions such as mineralization, nitrification, and fixation [71]. Concurrently, metagenomic and metatranscriptomic analyses show that rhizosphere and endophytic microbes highly express nitrogen-cycling genes (e.g., *nif*, *amo*, *nir*) under field conditions, directly mediating nitrogen transformations [72], while proteomic validation links gene potential to enzymatic activity, pinpointing rate-limiting steps in nitrogen metabolism [73].

**Figure 3:**
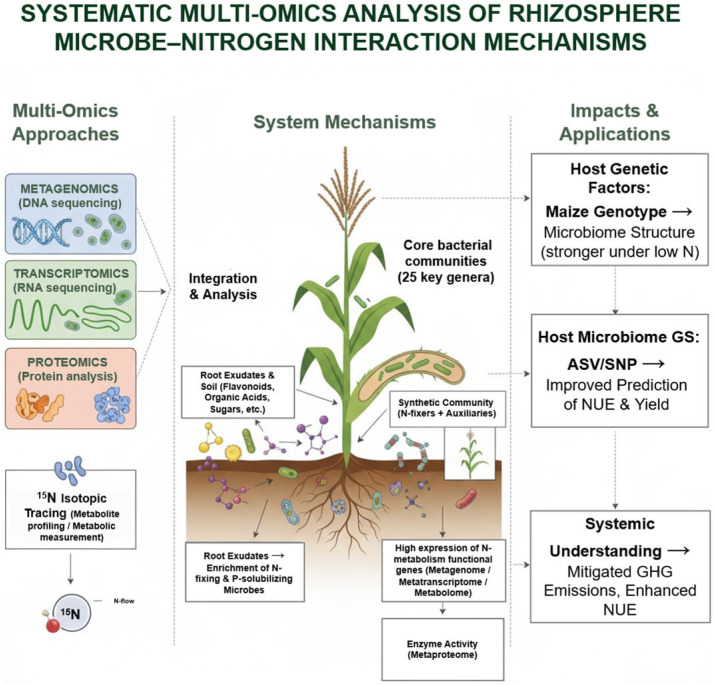
Multi-omics analysis of maize rhizosphere microbiome–nitrogen interactions. Integration of metagenomics, transcriptomics, proteomics, and isotopic tracing reveals how root exudates shape core microbial communities and synthetic consortia, driving high expression of nitrogen metabolism genes and enzyme activities. Host genetics and microbiome-guided selection influence microbiome composition and NUE, enabling systemic insights for improved yield, reduced N input, and mitigated greenhouse gas emissions.

Collectively, these multi-omics studies not only advance mechanistic understanding of maize microbiome–nitrogen interactions but also provide a robust framework for improving NUE, reducing fertilizer inputs, and mitigating environmental impacts (Figure 3). This integrative perspective underscores the potential of combining host genetics, microbiome engineering, and field-level management to achieve sustainable maize production.

## 6. Microbiome-Based Intervention Strategies to Enhance Nitrogen Acquisition

In the pursuit of improving nitrogen use efficiency, microbiome-based interventions have emerged as a frontier in applied research. Central to this approach is the identification and functional characterization of beneficial microbial strains. Numerous studies have reported diazotrophic bacteria capable of enhancing NUE, including *Azospirillum*, *Herbaspirillum*, and *Klebsiella*, all of which harbor complete *nif* gene clusters and can substantially supplement plant nitrogen under field conditions [16,22]. Additionally, arbuscular mycorrhizal fungi (AMF) establish mutualistic associations with the host, promoting the expression of nitrogen transporters, such as ZmAMT3;1, thereby enhancing inter-tissue nitrogen translocation efficiency [20]. Notably, core taxa such as Massilia have been recognized as pivotal contributors to NUE improvement, given their tight association with root exudate metabolism and potential influence on flowering time and yield formation [60,66]. Building upon these functional strains, the development of microbial fertilizers and bioinoculants has become an important practical avenue for enhancing NUE. Bioinoculants containing nitrogen-fixing bacteria can partially replace chemical nitrogen fertilizers, whereas composite microbial formulations—including diazotrophs, phosphate-solubilizing bacteria, and plant growth-promoting rhizobacteria—exhibit synergistic effects on multi-nutrient utilization [21,52]. Field trials indicate that a 20–30% reduction in nitrogen application, combined with microbial fertilizers, can maintain or even increase yield, demonstrating considerable potential for agricultural application. However, the performance of these inoculants is highly context-dependent, varying with soil type and climatic conditions, underscoring the need for site-specific optimization [48].

**Figure 4:**
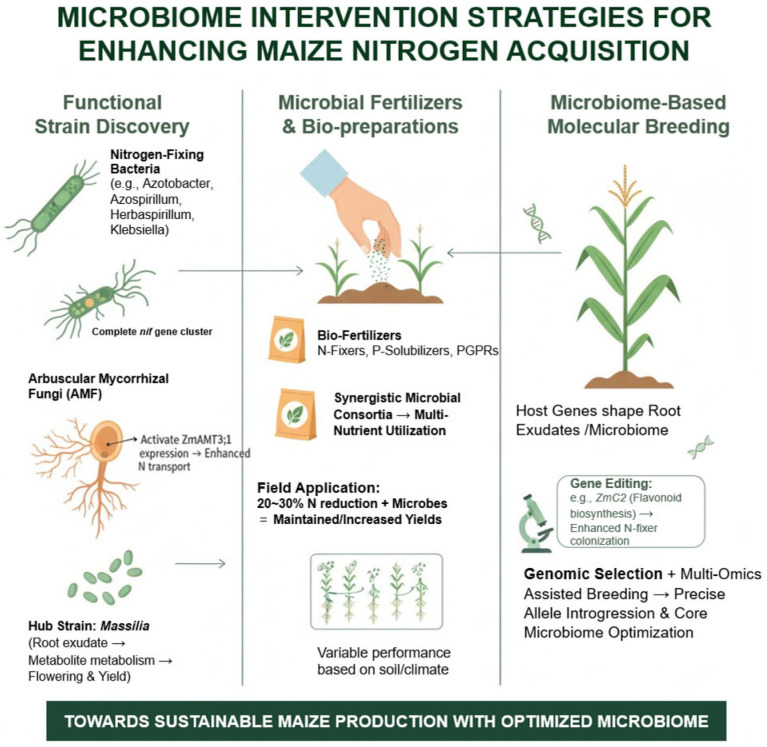
Microbiome-based strategies to enhance maize nitrogen acquisition. Three complementary approaches are illustrated: (1) Functional strain discovery: identification of N-fixing bacteria, AMF, and key hub strains that modulate root exudates to improve growth and yield; (2) Microbial fertilizers and bio-preparations: bio-fertilizers and synergistic microbial mixtures that enhance multi-nutrient utilization and can reduce N input by 20~30% under field conditions; (3) Microbiome molecular breeding: host genes and multi-omics-guided selection (including gene editing, e.g., *ZmC2*) optimize root exudates and core microbiomes for improved N acquisition. Together, these strategies aim to achieve sustainable maize production through an optimized rhizosphere microbiome.

A more forward-looking strategy involves microbiome-informed molecular breeding. Host genes dictate the composition and quantity of root exudates, thereby shaping rhizosphere microbial assemblages. For instance, the *ZmC2* gene regulates flavonoid biosynthesis, compounds that significantly enhance diazotroph colonization under low-nitrogen conditions [64]. Future approaches integrating gene editing to modify host–microbiome interaction genes, alongside genome-wide selection and multi-omics-assisted breeding, may enable simultaneous optimization of host genetics and core microbial communities, facilitating a systematic enhancement of NUE (Figure 4).

In parallel with microbial inoculants and microbiome-assisted breeding, biostimulants have recently emerged as a promising complementary strategy for enhancing nitrogen acquisition through modulation of rhizosphere microbial activity. Biostimulants derived from beneficial microbes, microbial metabolites, or organic compounds can stimulate native microbial communities, improve soil enzyme activity, and reshape root exudation patterns, thereby enhancing recruitment of diazotrophs, organic N mineralizers, and other nitrogen-cycling taxa. Importantly, biostimulants may act synergistically with host genetic mechanisms that regulate root exudates and microbial assembly (such as flavonoids controlled by *ZmC2* or mucilage secretion influenced by *ZmSBT3*), suggesting that integrating biostimulant application with microbiome-informed breeding could accelerate the development of cultivars with stable nitrogen-responsive microbiomes. Incorporating biostimulants into microbiome-based intervention frameworks, therefore, provides an additional, practical tool to enhance NUE while supporting long-term soil health and sustainable maize production.

## 7. Future Directions for Rhizosphere Microbiome-Mediated Regulation of NUE

**Figure 5:**
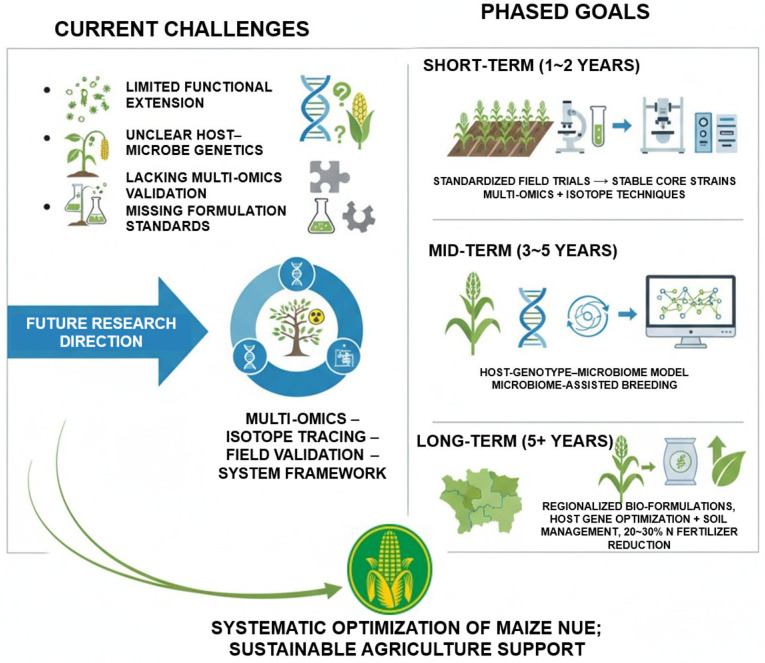
Future framework for rhizosphere microbiome-mediated enhancement of maize NUE. Integration of multi-omics analyses, isotopic tracing, and multi-site field validation links microbial functional potential to actual nitrogen transformation. Standardized microbial inoculants, predictive models incorporating host genotype and environmental factors, and microbiome-assisted breeding collectively aim to optimize nitrogen uptake, reduce fertilizer input, and achieve sustainable maize production.

Despite significant advances in elucidating maize-rhizosphere microbiome interactions through multi-omics approaches, translating mechanistic insights from controlled experiments to stable field applications remains challenging. Functional microbial strains and synthetic communities often exhibit variable performance across different soil types, climatic conditions, and native microbial backgrounds, limiting their reproducibility and scalability. Moreover, the genetic basis underlying host–microbiome interactions is still poorly understood. Although key host genes influencing microbiome recruitment, such as *ZmC2*, *ZmSBT3*, and *ZmNLP8*, have been identified, their effects show considerable plasticity across diverse genotypes and environmental contexts. Current metagenomic datasets largely reflect potential microbial functions, with limited integration of metatranscriptomics, metaproteomics, and isotopic tracing, resulting in incomplete functional validation. In parallel, standardized protocols for microbial inoculant formulation, production stability, and compatibility with chemical fertilizers remain underdeveloped, constraining large-scale application in agricultural systems (Figure 5).

Addressing these challenges requires a systematic framework centered on “multi-omics–isotope–field validation,” linking microbial functional potential to actual contributions to nitrogen transformation. In the short term, standardized multi-site field trials across diverse soil types should be conducted to identify core strains with stable field activity, accompanied by integrated multi-omics and isotopic workflows. In the medium term, predictive models incorporating host genotype, microbiome composition, and environmental factors should be developed, enabling the integration of rhizosphere microbial traits into genomic selection pipelines and facilitating microbiome-assisted breeding. Long-term goals include the establishment of region-specific microbial inoculant development and deployment systems, coupled with host genetic improvement, soil management, and precision fertilization, to achieve a 20~30% reduction in nitrogen input without yield penalties (Figure 5). Ultimately, the synergistic application of multi-omics approaches and microbiome interventions holds promise for systematically optimizing nitrogen use efficiency in maize across molecular to field scales, providing sustainable technological support for green agriculture.

## 8. Conclusions

This review highlights the rhizosphere microbiome as a key external driver of nitrogen use efficiency in maize. Microbial taxa involved in nitrogen fixation, mineralization, nitrification, and signaling interact with host genetic pathways regulating root architecture, nitrogen transport, and exudation. These interactions contribute substantially to plant nitrogen acquisition and can be shaped by host genotype. Microbial inoculants and synthetic communities show potential for enhancing NUE, while microbiome-informed genomic prediction models suggest that incorporating microbial traits into breeding pipelines can improve prediction accuracy. Nevertheless, the context-dependence of microbial functions and variable field performance of inoculants remain major challenges. Advancing maize NUE will require coordinated efforts to elucidate host–microbe interactions, identify robust microbial taxa, optimize microbiome-based interventions, and integrate microbial traits into breeding strategies. The rhizosphere microbiome thus represents both a valuable biological resource and a strategic target for sustainable, nitrogen-efficient maize production.

## Data Availability

No new data were created or analyzed in this study. Data sharing is not applicable to this article.
